# Current Insights into Phytochemistry, Nutritional, and Pharmacological Properties of *Prosopis* Plants

**DOI:** 10.1155/2022/2218029

**Published:** 2022-03-11

**Authors:** Jianshu Zhong, Peiyao Lu, Hanjing Wu, Ziyao Liu, Javad Sharifi-Rad, William N. Setzer, Hafiz A. R. Suleria

**Affiliations:** ^1^School of Agriculture and Food, Faculty of Veterinary and Agricultural Sciences, The University of Melbourne, Parkville, VIC 3010, Australia; ^2^Facultad de Medicina, Universidad del Azuay, Cuenca, Ecuador; ^3^Department of Chemistry, University of Alabama, Huntsville, Huntsville, AL 35899, USA; ^4^Aromatic Plant Research Center, 230 N 1200 E, Suite 100, Lehi, UT 84043, USA

## Abstract

*Prosopis* is a regional cash crop that is widely grown in arid, semiarid, tropical, and subtropical areas. Compared with other legume plants, *Prosopis* is underutilized and has great potentialities. *Prosopis* not only is a good source of timber, construction, fencing material, and gum, but also can be applied for food, beverage, feed, and medicine. *Prosopis* contains numerous phytochemical constituents, including carbohydrates, proteins, fatty acids, minerals, and vitamins, while varieties of phenolic compounds have also been identified from different parts of *Prosopis*. Flavonoids (especially *C*-glycosyl flavonoids), tannins, catechin, 4′-O-methyl-gallocatechin, mesquitol, and quercetin *O*-glycosides are significant phenolic contents in *Prosopis*. Various extracts of *Prosopis* displayed a wide range of biological properties, such as antioxidant, antihyperglycemic, antibacterial, anthelmintic, antitumor, and anticancer. Additionally, *Prosopis* has the potential to be an ideal diet that contains abundant dietary fiber, minerals, galactomannans, and low-fat content. However, the bioactivity and pharmacological properties associated with *Prosopis* were influenced by the bioavailability of phytochemicals, various antinutritional compounds, and the interactions of protein and phenolic compounds. The bioavailability of *Prosopis* is mainly affected by phenolic contents, especially catechin. The antinutritional compounds negatively affect the nutritional qualities of *Prosopis*, which can be prevented by heating. The protein-phenolic compound interactions can help the human body to absorb quercetin from *Prosopis*. This literature review aimed to provide systematic information on the physical, biochemical, pharmacological, and nutritional properties and potential applications of *Prosopis*.

## 1. Background

**Figure 1:**
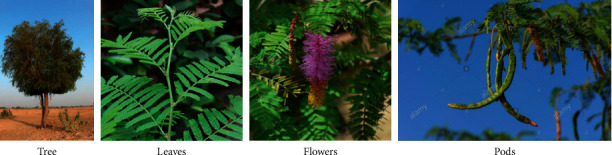
*Prosopis cineraria* tree, leaves, flowers, and pods [7].

*Prosopis* (*Prosopis* spp.) is an underutilized legume plant that belongs to the *Leguminosae* family and *Mimosaceae* subfamily [1]. *Prosopis* comprises 44 species, including *Prosopis juliflora*, *Prosopis farcta*, *Prosopis velutina*, *Prosopis glandulosa*, *Prosopis laevigata*, *Prosopis pallida*, and *Prosopis cineraria* [2]. Forty species of *Prosopis* come from North and South America, three are native to Asia, and the other one is from Africa [3]. *Prosopis* is mainly distributed in arid, semiarid, tropical, and subtropical countries, such as the United States, India, Argentina, Chile, Kenya, and Pakistan [4]. In South America, Argentina has the most varied *Prosopis*, with 29 species of 14 endemic taxa [4]. *Prosopis* grows widely from the southwestern part of the United States to the Argentinean Patagonia, which is an important characteristic of the Monte and Chaco desert region in Argentina from Salta to Chubut provinces [4]. The ecological value of *Prosopis* is due to their resistance to heat, drought, salinity, and alkalinity, while *Prosopis* can also promote nitrogen fixation to stabilize and improve the soil [5]. Furthermore, *Prosopis* does not need to be grown annually, while it can also be grown with other crop species, such as millet crop in India [6]. The leaves of *Prosopis cineraria* are glabrous or puberulous and deciduous, with a length of 2–7 cm [7]. The fruits are elongate, slender, and 10–21 cm long, while the peel is brittle and thin [7]. The pods of *Prosopis cineraria* consist of about 70% of the pericarp and 30% of the seeds, while the seeds are brown in color and ovate in shape [8]. The appearance of the *Prosopis cineraria* tree, leaves, flowers, and pods are demonstrated in Figure 1. *Prosopis* can be consumed as beverages, flour, sweets, jams, bread, cakes, cookies, and syrup [9]. The *Prosopis* flour has brown color and sweet flavor, with a similar aroma to coffee, cocoa, coconut, or caramel [10]. Furthermore, the *Prosopis* gum, which is exuded from the bark of the *Prosopis* tree, can be used as an emulsifier, film-forming agent, foaming agent, tablet binder, and stabilizer [5]. *Prosopis* also provides fruit, firewood, timber, livestock feed, vegetable, construction and fencing material, medicine, and shade [11]. *Prosopis* can be applied as a folk medicine for different diseases, while the decoction from its twigs and flowers has antidiabetic capacity. For example, leaf extracts of *Prosopis* had antibacterial, antihyperglycemic, antihyperlipidemic, and antioxidant properties [12]. Overall, as all the parts of the tree are useful, *Prosopis* is called kalpataru in India, which means the “wonder tree” and the “king of the desert” [11].

## 2. *Prosopis* Cultivation and Applications

*Prosopis* trees are widely distributed in the regions that have the features of an extremely arid climate, low and erratic rainfall, dry atmosphere, and high wind velocities [11]. *Prosopis* is suitable to grow in arid and semiarid conditions of the desert because it can absorb water from the groundwater resources at a length of about 20 meters by its well-developed and expansive tap root system [13]. Also, *Prosopis* plays a significant role in desert ecosystems in that it can be a biomass producer and can enrich desert soil, fix atmospheric nitrogen, and increase the rate of greening. It not only can promote ecological stability of the region, but also is a vital resource for human beings, livestock, and soils [14]. *Prosopis* can tolerate extreme temperatures, alkalinity, and salinity, while it does not require annual plantings and can grow with other crop species, such as millet crop in India. However, the economic importance of *Prosopis* in the global market is limited, largely due to the ignorance of researchers and industry. The reason is that the *Prosopis* is presently only a regional cash crop and is not as important at the global level [15].

A *Prosopis* tree can yield approximately 20 to 50 kg·yr^−1^ of pods. If the *Prosopis* tree is cultivated as an orchard crop, the production can range from 1 to 8 t·ha^−1^·yr^−1^ of pods, while the production of *Prosopis* can yield more than 10 t·ha^−1^ yr^−1^ [16]. Furthermore, *Prosopis* gum is mainly produced in Mexico [5]. The main source of *Prosopis velutina* gum is the desert plains of the northwestern state of Sonora in Mexico, while the *Prosopis laevigata* gum is mainly collected from the lowlands of the northeastern state of San Luis Potosi (Mudgil and Barak, 2020) [5]. In Sonora, *Prosopis* gum is known as chucata, while its production starts in May to July and ends in July to August. *Prosopis* gum is collected by hand in a low humid environment, high temperature, and spiny vegetation. It is reported that the average tree density in the central region of Sonora is more than 80 trees/ha, while only approximately 8 trees/ha are gum producers [5].

*Prosopis* wood is suitable to make furniture and parquet flooring, due to its hardness, durableness, and appealing color. *Prosopis* wood can be also applied for construction, charcoal production, and mulch in gardens [3]. In addition, the bark of *Prosopis* is used as an astringent, tonic, and blood purifier and is said to be a potent drug for several ailments such as leprosy, dysentery, bronchitis, asthma, leucoderma, piles, muscular tremors, asthma, rheumatism, and inflammations [17]. The traditional medicines in India use *Prosopis* wood ash to treat gastrointestinal, respiratory, and cardiovascular diseases [11].

*Prosopis* leaves have antioxidant and hypoglycemic capacity due to their sterols contents, including campesterol, stigmasterol, and octacosanol [18]. The leaves contain an amount of protein, carbohydrates, and mineral matter and have high nutritive value [17]. The methanol leaf extract of *Prosopis* not only can reduce tumor growth, but also present in vitro and in vivo anticancer ability [19]. The smoke of the *Prosopis* leaves can be applied for treating eye sickness [20]. Leaf paste can be used to treat boils and blisters, such as mouth ulcers in livestock and leaf infusion on open sores on the skin [17]. Furthermore, *Prosopis* leaves can be the feed for camels, goats, and donkeys [11].

As an exudate from the tree rind, the *Prosopis* gum has several characteristics including high solubility, emulsifying, and high viscosity, which are similar to Arabic gum, and can act as a kind of stabilizer [21]. First, *Prosopis* gum can contribute to preparing emulsion and improving the stability of the emulsion, and therefore, *Prosopis* gum can function as an emulsifier [5]. The properties of *Prosopis* gum are similar to Arabic gum that can contribute to the formation and stabilization of oil-in-water emulsion and exhibit potential for the encapsulation of orange citrus oil for the improvement of spray-dried products. However, compared with Arabic gum, *Prosopis* gum is a better emulsion stabilizer because it has unique advantages in providing more stability and lower mean size of oil droplets [22]. Second, *Prosopis* gum can be used as a replacer of Arabic gum to encapsulate the food to maintain colors, aroma, and flavors through spray drying [5]. *Prosopis* gum not only can encapsulate alone but also can be used in association with other encapsulating agents to encapsulate essential oils. Third, *Prosopis* gum is used as a foaming agent due to its foaming properties [5]. Forth, the addition of *Prosopis* gum can increase the shelf life of food [5]. For example, because of the chemical composition, *Prosopis* gum can be added to bread to increase the shelf life to 10 days. Finally, edible films made by *Prosopis* gum are excellent barriers to prevent oxygen, water vapor, and CO_2_ so that they can develop storage stability and extend the shelf life of fruits and vegetables [23].

*Prosopis* pods are comprised of approximately 72% pericarp and 28% seeds. The seeds of *Prosopis* pods consist of episperm, endosperm, and cotyledons, while the pericarp is constituted by epicarp, mesocarp, and endocarp [24]. Carbohydrates (including fiber and soluble sugars), proteins, and bioactive compounds, such as tannins, steroids, flavones, and alkaloids, are the main components of *Prosopis* pods, which provide *Prosopis* pods with antioxidant, anti-inflammatory, and antihypertensive functions [10]. With the advantages of rich energy and high nutritional values, the *Prosopis* pods are a good source of food for human beings [11]. *Prosopis* pods are considered delicious, fragrant, and sweet [9]. This is the main reason that *Prosopis* pods can be used in a variety of foods such as bread, cakes, cookies, jams, syrup, beverages, fermented beverages, “atoles” (Mexican beverage), cheese, and a coffee or chocolate substitute [9,25]. Besides, in South America, “añapa,” which is a kind of sweet, non-fermented, and nonalcoholic beverage, and “aloja,” which is a fermented beverage with alcohol, are two popular food products produced by *Prosopis* pods [26]. People in South America also grind, roast *Prosopis* pods, or mix with milk to produce flour, cakes, and syrup [26]. *Prosopis* pods are also used to make a dessert called “bolanchao,” which is popular in Argentina [24]. The syrup derived from the *Prosopis* pods is also commercialized in South American countries, such as Peru, Ecuador, and Chile, for making confectionery and cocktails [27]. The water extraction of the sugars from the *Prosopis* pods can be used to make a beverage called “yupisín,” while “algarrobina” is the syrup boiled from *Prosopis* pods [16]. Furthermore, it is popular to use dried *Prosopis* as animal feed [17]. With the right proportion, *Prosopis* pods can be effectively used in the animal feed industry [28]. Without affecting product quality, feed intake, feed conversion, and body weight gain of Omani goats can be increased by the addition of 20% or 200 g/kg *P. juliflora* pods in feed [28]. Furthermore, in Ethiopia, 10 and 20% inclusion of ground *P. juliflora* pods were found to reduce feed production costs without influencing biological performance [28].

*Prosopis* flour (PF) has brown color and sweet taste, while it has roughly the same energy and protein content as wheat flour. Furthermore, this gluten-free flour also has flavors of coffee, cocoa, coconut, caramel or molasses, cinnamon, and hazelnut [9,29]. The *Prosopis* flour, which is called “mesquite flour” or “algarroba flour,” is made by the whole ripe *Prosopis* pods [10]. It has been studied that *Prosopis* flour can be applied for composite bread and other cereal-based formulations because *Prosopis* flour is rich in iron, potassium, fiber, and calcium, and the amino acids in *Prosopis* flour complement cereal proteins [10,30]. The *Prosopis* flour levels are limited in bread formulations by the lack of starch. The mixture of 5–25% *Prosopis* flour and wheat flour can develop the taste of food products; 5% *Prosopis* flour is suitable to add in sweet bread to gain acceptable taste and texture, while 25% *Prosopis* flour is applied for producing biscuits, which can reduce the addition of sugar [16]. The distinct viscoelastic characteristics of wheat dough of *Prosopis* are related to the gluten network [9]. The addition of *Prosopis* flour makes a negative influence on the volume, texture, and structure of bread since it cannot only disturb the gluten network but also result in network deficiency and different rheological behavior due to the existence of the globular proteins and fiber in *Prosopis* flour. Besides, there are technical problems such as shredded crumbs and low volume that are caused by the gluten-free properties of *Prosopis* flour. Furthermore, with the content of *Prosopis* flour increased, the maximum volume, specific volume, and compact crumb would decrease, while the fermentation times would increase. As a result, the major difficulties to add *Prosopis* into baked food are the rheological changes in the dough and the final product [9].

## 3. Phytochemicals in Prosopis

The main nutrient components of *Prosopis* are shown in Table 1. The methods and technologies, which were used to determine the major chemical compounds in *Prosopis*, are demonstrated in Table 2. The identification of phytochemicals in *Prosopis* is summarized and displayed in Table 3.

### 3.1. Nutritional Composition

The major nutrient components of *Prosopis* include macronutrients (carbohydrate, fat, protein, amino acids, fatty acids, and fibers), micronutrients (minerals), and phytochemicals (polyphenolic compounds, carotenoids, and ascorbic acid) [31]. The *Prosopis* pods contain 7 to 22% protein, 30 to 75% carbohydrates, 11 to 35% crude fiber, 1 to 6% fat, 3 to 6% ash, 0.33% calcium, and 0.44% phosphorus [16]. Also, the raw seeds of *Prosopis* were comprised of 11% moisture content and 89% dry matter (including 39% protein, 4.5% fat, 18.5% carbohydrates, and 4% ash) [17].

### 3.2. Macronutrients

#### 3.2.1. Carbohydrate, Fat, and Protein

The main components of carbohydrates in *Prosopis* are galactose and mannose, while *Prosopis* also contains minor amounts of glucose and arabinose, which might be caused by a complex polysaccharide composition or contaminants proceeding from the seed coat of *Prosopis*. In *Prosopis flexuosa* seed endosperm, galactomannan is the major polysaccharide (about 85% w/w of galactose plus mannose) [32].

*Prosopis* pods contain varieties of macronutrients, while different parts of *Prosopis* pods have different macronutrient contents. The carbohydrate content of cotyledon flour of *Prosopis alba* pods was 8.97 ± 0.05 g/100 pod flour, which was significantly lower than that of mesocarp pod flour (52.08 ± 0.09 g/100) [1]. Besides, the content of soluble reducing sugar in cotyledon flour (0.21 ± 0.07 g/100g) was also lower than that of mesocarp flour (3.73 g/100 g pod flour), while the fat content in cotyledon flour was higher with the value of 12.20 ± 0.05 g/100 g flour [1]. The main content in cotyledon flour of *P. alba* pods was crude protein (62.09%), and by comparison, the crude protein in mesocarp flour was only lower than 4% [1]. Furthermore, the protein content in cotyledon flour of *P. alba* pods was significantly higher than that in soybeans (34.6%), lentils (25.4%), peas (22.9%), and chickpeas (18.5%) [31]. The main protein varieties in cotyledon flour of *P. alba* pods were albumin (44.59 ± 1.70%) and globulin (30.82 ± 1.64%) [31]. Overall, with the advantages of high protein content with biological capacity, low carbohydrate content, and low-fat content, the cotyledon flour of *Prosopis alba* can be applied for producing foods or food supplements with low calories, which can be also along with the addition of cereal proteins (such as cookies, cereal bars, and coffee substitutes) [33].

*Prosopis* gum is a complex carbohydrate that consists of D-galactose, L-arabinose, D-glucuronate, D-mannose, and D-xylose, but it does not contain L-rhamnose, which makes it different from Arabic gum in structure [5]. In addition to carbohydrates, *Prosopis* gum also contains about 3% protein [34]. However, mesquite gum obtained from *Prosopis alba* gum contains more than 13% protein, which is much more than that of other *Prosopis* [35]. Serine, hydroxyproline, valine, and glycine are the main amino acids of protein in *Prosopis* gum [36]. It is reported that the emulsification ability of *Prosopis* gum is chiefly related to the protein content, and therefore, *Prosopis alba* gum has a stronger emulsification ability [37]. Fatty acids including palmitic, stearic, and behenic acid are also significant contents in the lipid portion of *Prosopis* gum [5].

#### 3.2.2. Amino Acids

Animal proteins are usually complete proteins with complete essential amino acids, and most plant proteins (including cereal proteins) are incomplete proteins, which lack one or more essential amino acids [31]. However, *Prosopis* flour contained all essential amino acids. Several essential amino acids, such as isoleucine and valine, which were deficient in other grains, were rich in concentration in *Prosopis* flour [31]. *Prosopis* flour also contains amino acids with sulfur (cys 1.47%), which is also limited in other grains [31]. The previous study demonstrated that the dry matter of *P. juliflora* pods consists of 0.99% aspartic acid, 0.28% threonine, 0.14% cystine, 0.43% valine, 0.10% methionine, 0.27% isoleucine, 0.52% leucine, 0.29% tyrosine, 0.33% phenylalanine, 0.37% alanine, 0.19% histidine, 0.32% lysine, 0.56% arginine, 0.41% serine, 1.4% glutamic acid, and 0.51% glycine (Rani et al., 2013). Based on the previous study, the main amino acids in *Prosopis* were Asp and Glu, which took up about 30% of the total amino acids with the value of approximately 22 mg/100 g protein [38]. The content of another amino acid, Leu, was about 6.5 mg/100 g protein [38]. Besides, the total essential amino acids (TEAAs) take up more than 45% with the value of 34 mg/100 g protein in *Prosopis alba*, which is similar to the values of two oil seeds, *Vigna subterranean* and *Phaseolus coccineus* [39,40]. The nutritional value of a protein depends on the nitrogen content and essential amino acids, and thus, *Prosopis* has the potential to provide essential amino acids to people as a diet [38]. The hydrophobic region of the protein in *Prosopis* was comprised of 6.5 g/100 g protein for an essential aliphatic amino acid (EAAA), which meant that *Prosopis* had good emulsification capacity [38]. Furthermore, the content of the total acidic amino acid (TEAA) (31.3 g%) was higher than the total basic amino acid (TBAA) (15.7 g%), which demonstrated that the protein in *Prosopis* is acidic [38].

#### 3.2.3. Fatty Acids

The most abundant fatty acid in *Prosopis* was palmitic acid, which was followed by oleic acids [10]. However, *Prosopis* only contained very little caprylic acid, capric acid, lauric acid, and pentadecanoic acid. In the previous study, nine fatty acids in cotyledon flour of *Prosopis alba* were analyzed, which included saturated fatty acids (SFAs), monounsaturated (MUFAs), and polyunsaturated fatty acids (PUFAs) [31]. As a result, PUFA (linoleic acid, 60%) was the major total fatty acid (TFA) in *Prosopis alba*, which was followed by MUFA (oleic acid, 18%) and SFA (palmitic acid, 16%). As an essential fatty acid, linoleic acid (omega-6 fatty acids) cannot be synthesized by humans and other animals, which can be only gained from food sources [31]. Linoleic acids play an important role in people's health. First, linoleic acids can regulate renal and pulmonary function, vascular tone, and inflammatory responses by functioning as structural components of membranes and precursors of eicosanoids [5]. Second, linoleic acid not only can induce the expression of uncoupling proteins and coordinately up modulate different dozen genes related to oxidative energy metabolism but also can motivate mitochondrial biogenesis and increase energy reserves, which causes stabilized synaptic properties [41]. Therefore, *Prosopis* has the potential to be a good source of essential fatty acids. Furthermore, it was also reported that *Prosopis* flour can be also applied for composite bread to increase the content of healthy unsaturated fatty acids. Additionally, the fatty acid profile in bread was not influenced by the heating process [42].

#### 3.2.4. Fiber

*Prosopis alba* flour contained more than 9% insoluble fiber, so it can be a good source of fiber [31]. Cellulose, hemicellulose, lignin, extractives, ash, and water are the main content of woody biomass. Based on a previous study, *Prosopis juliflora* contains 25%–30% hemicellulose, 40%–45% cellulose, 11%–28% lignin, and 3%–15% extractives [43]. *Prosopis* gum is a great source of dietary fiber due to its low viscosity, which is widely used in producing fiber-fortified food products [5]. The low viscosity of complex carbohydrates is very much suitable for its use as a dietary fiber source. Dietary fibers are complex carbohydrates that are resistant to enzymatic secretion of the human gastrointestinal tract and are beneficial for human health [16].

### 3.3. Micronutrients

#### 3.3.1. Minerals

Minerals play an important role in mental and physical health because they are vital constituents of bones, teeth, soft tissues, hemoglobin, muscle, blood, and nerve cells [31]. The richest mineral in the cotyledon flour of *Prosopis alba* was potassium (7.5 mg/g flour), while the sodium content was low (0.09 mg/g flour), which made *Prosopis* cotyledon flour contribute to balance electrolyte in diets [31]. The cotyledon flour of *Prosopis alba* is suitable to be used in the diets of people who need to control hypertension by having diuretics. Cotyledon flour of *Prosopis alba* also contained 1.07 mg/g flour of Ca and 0.10 mg/g flour of Fe. In addition, 100 g cotyledon flour of *Prosopis* can provide 90% of the daily requirements of Mg (3.0 mg/g flour) and 70% of the daily requirements of P (9.0 mg/g flour) [31]. Furthermore, the seeds of *Prosopis* were rich in potassium, magnesium, calcium, and phosphorus, while the values of copper, zinc, iron, and manganese were low. The mineral content in the *Prosopis* flour was similar to soybean, cowpea, African yam bean, and *Triticum durum*, which meant that *Prosopis* flour can be applied for feed supplement. The ratio of sodium to potassium, Na/K, was 0.18, which demonstrated that *Prosopis* can help to reduce high blood pressure. The Ca/P ratio of *Prosopis* was 3.76, which showed that *Prosopis* had the potential to be a good source of minerals for bone formation [38].

### 3.4. Phytochemicals

#### 3.4.1. Phenolic Compounds

70% aqueous/methanol was used to extract the free polyphenols of cotyledon flour of *Prosopis alba* [1]. The polyphenol content of the extract was 1150 ± 20 mg GAE/100 g flour, which was higher than that of mesocarp flour of *Prosopis* (180 to 410 mg GAE/100 g flour) and white wheat flour (4.4–14 mg GAE/100 g flour) [4]. Furthermore, cotyledon flour of *Prosopis alba* contained similar free phenolic compounds to flour of argentine native plants *Ziziphus mistol* (1190 ± 68 mg GAE/100 g DW) and *Geoffroea decorticans* (1240 ± 30 mg GAE/100 g DW) [1,44,45]. Furthermore, the polyphenolic content in fruits can be divided into low (<100 mg GAE/100 g), medium (100–500 mg GAE/100 g), and high (>500 mg GAE/100 g) types, and thus, cotyledon flour of *Prosopis alba* had high polyphenolic content [45].

**Figure 2:**
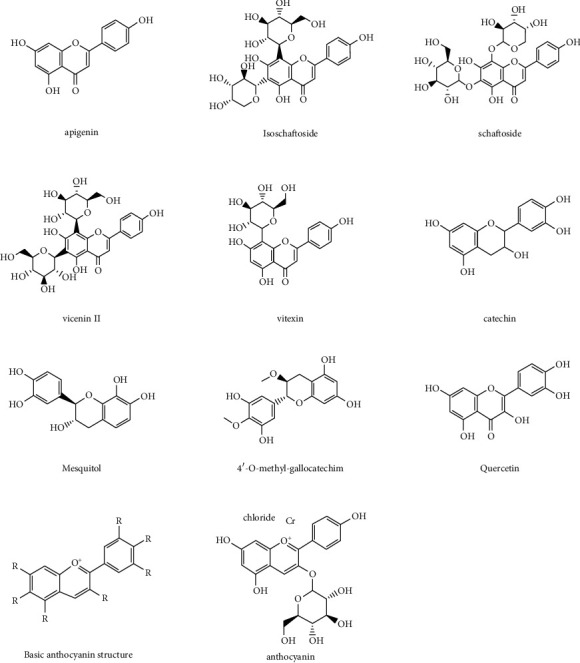
Chemical structures of some selected phenolic compounds extracted from *Prosopis* plants.

In the cotyledon flour of *Prosopis alba*, *C*-glycosyl flavonoids were the major free phenolic compounds, which were followed by tannins [4]. Flavonoids are a significant type of polyphenols that commonly exist in vegetables and contribute to maintaining human health, and therefore, they have attracted more attention than before [46,47]. The previous study showed that flavonoids could be the inhibitors of glycohydrolases. The high galactomannan content may positively influence the accumulation of *C*-glycosidic flavones in *Prosopis* seeds [48]. Apigenin *C*-glycosides are stronger *a*-glucosidase inhibitors than acarbose, and therefore, *C*-glycosides are potential to apply for delaying the absorption of carbohydrates [26]. Besides, tannins are another important polyphenolic class because of their structural diversity and bioactivity [49]. Flavonoid content was also high in the mesocarp flour, while cotyledon flour of *Prosopis alba* did not contain hydrolysable tannins and anthocyanins [31]. Polar extracts from *P. juliflora* wood and bark contained several flavanols, including catechin, 4′-O-methyl-gallocatechin, and mesquitol [50]. Quercetin *O*-glycosides, apigenin-based *C*-glycosides, flavonoid *C*-glycosides, and *O*-glycosides are also important phenolic compounds in *Prosopis*, which present anti-inflammatory, antioxidant, and antiplatelet properties [4,46]. The structures of some typical phenolic compounds mentioned above are shown in Figure 2. By balancing antioxidants and oxidants in the human body, phenolic compounds can control oxidative stress and prevent chronic illnesses, such as cancer, atherosclerosis, or cardiovascular disease [45].

#### 3.4.2. Carotenoids and Ascorbic Acid

Both carotenoids, which can be precursors of vitamin A, and ascorbic acid are good antioxidants. Furthermore, carotenoids and ascorbic acid can delay the formation of off-flavors and rancidity compounds to extend the shelf life of the product, so they are applied for natural food or beverage preservatives [31]. In *Prosopis* flour, the carotenoid content was 10.55 ± 0.05 mg-CE/100 g flour, which was significantly higher than ascorbic acid (0.33 mg L-AA/100 g flour) [31]. As essential structural contents of the photosynthetic and reaction center, carotenoids can protect photosynthetic organisms to avoid potentially harmful photooxidative processes [51]. Besides, carotenoids are a class of natural fat-soluble pigments found principally in plants, algae, and photosynthetic bacteria, where they contribute to the photosynthetic process. Furthermore, carotenoids can also act as accessory pigments and protect chlorophyll from photooxidative destruction [52].

## 4. The Pharmacological Value of Prosopis

*Prosopis* is widely used as a folk medicine for a variety of ailments in South America [7]. *Prosopis* flowers can be applied to safeguard against miscarriage during pregnancy by pounded and mixed with sugar [17]. The *Prosopis* flowers with twigs can be used as an antidiabetic agent [7]. With the features of dry, acrid, and bitter with a sharp taste, the bark of *Prosopis* cineraria trees can be applied for treating leprosy, dysentery, rheumatism, bronchitis, asthma, cough colds, leucoderma, muscle tremors, and piles as an internal drug [17]. It was also reported that the bark of *Prosopis cineraria* trees has anti-inflammatory activity and can cure scorpion sting [17]. The smoke of *Prosopis* leaves can be used for treating eye infections [7]. The dry pods of *Prosopis* also contribute to avoiding protein-calorie malnutrition and iron calcium deficiency in the blood [7]. Different extracts of *Prosopis* demonstrate various pharmacological activities, such as analgesic, antipyretic, antihyperglycemic, antioxidant, antihypercholesterolemic, and antitumor, which will be discussed in the following paragraphs. The bioactivities of phytochemical compounds isolated from *Prosopis* are displayed in Table 4.

### 4.1. Antioxidant Activity

DPPH, ABTS, hydroxyl, superoxide, nitric oxide, and hydrogen peroxide were used to estimate the antioxidant potential of different leaf solvents of *Prosopis laevigata* in a previous study [11]. The results demonstrated that the content in *Prosopis laevigata* leaves can provide hydrogen and remove an odd electron from a free radical to avoid radical reactivity. Besides, methanolic and ethyl acetate extracts have the strongest scavenging capacity [11].

According to ABTS radical assay, *n*-hexane, methylene chloride, ethyl acetate, and *n*-butanol extracts of *Prosopis farcta* aerial parts presented 83.1, 82.0, 87.2, and 87.0% inhibition percentages (I%), respectively, compared with ascorbic acid (89.2%) [54]. The extract of *P. farcta* fruit also showed similar results to *P. farcta* aerial parts, while the antioxidant capacity was directly related to the high phenol and flavonoid contents [55]. Flavonoids are important natural antioxidants that can be obtained from *Prosopis* [56]. In both *in vitro* and *in vivo* conditions, the extracts of *Prosopis* were detected to have antioxidant capacity due to the content of flavonoids, while the antioxidant properties *in vitro* were higher than those *in vivo*. However, if the state of oxidation is not induced, a high concentration of flavonoids in the extract of *Prosopis* can have a prooxidant effect in an *in vivo* environment [43]. Additionally, the methanolic extract of the *Prosopis farcta* stem bark also had antioxidant activity [57]. The methyl linoleate oxidation inhibition test was also applied for evaluating the antioxidant capacity of *Prosopis juliflora* extract [43]. As a result, the oxidation of methyl linoleate induced by 2, 2′-azobisisobutyronitrile can be inhibited by (+)-catechin and (-)-mesquitol in *Prosopis* extract. Besides, the antioxidant capacity of the flavanol extract of *Prosopis juliflora* was stronger than that of butylated hydroxytoluene (the reference antioxidant), probucol, and *a*-tocopherol [58]. Overall, *Prosopis* has the potential to be a source of natural antioxidants for food supplements or pharmaceutical industry formulations and can be used in treating inflammatory diseases, cancer, and diabetes [59].

### 4.2. Antihyperglycemic Activity

Alloxan-induced hyperglycemia model was applied for evaluating the antihyperglycemic capacity of 50% hydroalcoholic extract of stem bark of *Prosopis cineraria* [11]. A group of hyperglycemic mice was given 300 mg/kg B W once a day for 1.5 months. As a result, the body weight and fasting blood glucose level of those mice were decreased, while liver glycogen content was increased compared with the control group. Furthermore, the *Prosopis* could also increase the activity of antioxidant enzymes and the concentration of nonenzymatic antioxidants to reduce the oxidative damage in the tissues of hyperglycemic mice [60]. Therefore, the *Prosopis cineraria* extract has antidiabetic and antioxidant potential.

### 4.3. Analgesic and Antipyretic Activity

Using Brewer's yeast-induced hyperpyrexia model, the petroleum ether extract of stem bark of *Prosopis cineraria* showed antipyretic capacity in experimental rats [7]. The ethanolic extract of the root of *Prosopis cineraria* was determined by tail immersion and hot plate method [61]. In addition, an acetic acid-induced writhing test model was applied for evaluating the analgesic activity of the aqueous extract of *Prosopis cineraria* leaves [7]. As a result, the dose of 200 mg/Kg B W can develop analgesic activity in mice [61]. Furthermore, through Brewer's yeast-induced hyperpyrexia model, the antipyretic activity was also developed at the same dose [11].

### 4.4. Antitumor Activity

Hydroalcoholic extract of *Prosopis cineraria* leaves and bark demonstrated antitumor properties against the Ehrlich ascites carcinoma tumor model [7]. Also, the protective action against induced experimental liver tumors of methanolic extract of *Prosopis cineraria* leaves was estimated in male Wistar rats. The administration of the extract (200 and 400 mg/kg) can decrease the levels of mitochondrial lipid peroxidation (LPO) and liver weight in a dose-dependent manner, while the levels of mitochondrial enzymatic antioxidant can also be reduced by the extract [7].

### 4.5. Respiratory and Gastrointestinal Activity

The spasmolytic, bronchodilator, and vasodilator properties of methanolic extract from the stem bark of *Prosopis cineraria* were determined in a previous study [7]. As the result, blockade of Ca^2+^ channels may mediate the bronchodilator and vasodilator activities.

### 4.6. Anticonvulsant Activity

Through inducing convulsions in mice, pentylenetetrazole (PTZ) and maximal electroshock (MES) can be used to estimate the anticonvulsant activity of methanolic extract of *Prosopis cineraria* [11]. As a result, methanolic extract of stem barks of *Prosopis cineraria* had an anticonvulsant capacity, while the hind limb tonic extensions (HLTEs) induced by MES can be inhibited and the drug against PTZ-induced seizures can be protected by the *Prosopis* extract. The duration of convulsions can be decreased by the mixture of methanolic extract of *P. cineraria* at doses of 200 and 400 mg/kg and phenytoin at 25 mg/kg. Overall, the inhibition of MES-induced seizures and the protector effect of drugs against PTZ-induced seizures can play important role in myoclonic control and the absence of seizure protection to human beings [62].

### 4.7. Antibacterial Activity

It is important to seek new antibacterial compounds due to microbial antibiotic resistance [3]. It is reported that juliflorine, synthesized in *Prosopis*, has antibacterial capacity against *Corynebacterium diphtheria* var. *mitis*, *Corynebacterium hoffmanni*, *Bacillus subtilis*, *Staphylococcus aureus*, and even *Streptococcus pyogenes*, which is resistant to most antibiotics [63]. Besides, varieties of alkaloids, tannins, phenols, flavonoids, terpenes, and steroids can be obtained from different parts of *P. juliflora*, which have antibacterial capacity against Gram-negative bacteria [3]. These Gram-negative bacteria are resistant to antibiotics such as minocycline, chloramphenicol, and erythromycin.

In a previous study, the potential antibacterial capacity of aqueous and ethanolic extract of *P. farcta* against *methicillin-resistant S. aureus* (MRSA) was determined. As a result, the minimum inhibitory concentration (MIC), which means the least concentration of antimicrobial agent that prevents microbial growth, of aqueous extract was 100 mg/mL, while the MIC of ethanolic extract was 25 mg/mL [64, 65]. For comparison, previous research on the antimicrobial effect of tomato seeds indicated that the MIC of the two types of tomato seeds remained essentially in the range of 5–20 mg/mL when extracted with organic solvents, notably both types of tomato seeds extracted with hexane having MIC values of 5 mg/mL [66].

Another antibacterial research on brown seaweed extraction indicated the MIC of the several seaweeds analyzed was generally higher than or equal to 31.3 mg/ml for MRSA [67]. Both of the research above presented favourable results in terms of antibacterial capacity.

Besides, the minimum bactericidal concentration (MBC), which means the least concentration of antimicrobial agent required to kill microorganisms, of aqueous and ethanolic extracts was 25 and 12.5 mg/mL, respectively [64, 65]. The *n*-hexane and methylene chloride extract of *P. farcta* aerial parts showed moderate antimicrobial activity. The n-hexane extract exhibited capacity against *Shigella* spp., *Escherichia coli,* and *Proteus vulgaris* with inhibition zones of 4.7, 8.3, and 6.3 mm, respectively, compared with streptomycin and ampicillin as standard antibiotics (with inhibition zones of 14, 24, and 18 mm, respectively) [68]. The methylene chloride extract can prevent against *Erwinia* spp., *Escherichia coli,* and *Staphylococcus epidermis* with inhibition zones of 6.2, 7.2, and 8.4 mm, respectively, compared with streptomycin and ampicillin as standard antibiotics (with inhibition zones of 35 and 24 mm, respectively) [68]. Besides, the ethyl acetate extract showed antimicrobial activity to inhibit *Shigella* spp., *Escherichia coli*, and *Candida albicans* with inhibition zones of 7.3, 11, 6, and 7.3 mm, respectively, compared with streptomycin, ampicillin, and clotrimazole as standard (with inhibition zones of 14, 24, 18, and 20 mm, respectively) [68]. Furthermore, *n*-butanol extract displayed the capacity to inhibit *Shigella* spp., *Erwinia* spp., *Escherichia coli*, *Proteus vulgaris*, *Staphylococcus epidermis,* and *Candida albicans* with inhibition zones of 11, 9, 17, 12.4, 9.7, and 11 mm, respectively, compared with streptomycin, ampicillin, and clotrimazole as standard (with inhibition zones 14, 35, 24, 18, 24, and 20 mm, respectively) [57].

Agar well diffusion method can be used to estimate the antimicrobial properties of extracts of different parts (pods and steam bark) of *Prosopis cineraria* [11]. For *Prosopis cineraria* pods, methanol extract can inhibit *E. coli*, *P. aeruginosa*, *S. typhi*, and *K. pneumoniae*, but chloroform and aqueous extracts cannot inhibit those microorganisms. Besides, for *Prosopis cineraria* stem bark, the methanolic and aqueous extracts demonstrated moderate antibacterial capacity to *E. coli*, *P. aeruginosa*, *S. typhi*, and *K. pneumoniae*, which is related to the flavonoid and tannin content [69].

The 20% aqueous extract of *Prosopis juliflora* leaves also had antibacterial activity against three different phytopathogenic *Xanthomonas* pathovars, namely *Xanthomonas axonopodis* cf. *phaseoli* with an inhibition zone of 12.07 ± 0.14 nm, *Xanthomonas axonopodis* cf*. malvacearum* with an inhibition zone of 15.92 ± 0.07 nm, and *Xanthomonas campestris* cf. *vesicatoria* with an inhibition zone of 16.37 ± 0.12 nm [43,70]. These pathogenic bacteria can cause common blight of beans, angular leaf spot of cotton, and bacterial spot of tomato. Furthermore, 14 human pathogenic bacteria were also inhibited by the *Prosopis* extract. Likewise, the *Prosopis juliflora* extract had an antidermatophytic capacity that could be used to inhibit *Trichophyton mentagrophytes* infection in rabbits [70]. As a result, 2.5% juliflorine obtained from *Prosopis juliflora* can cure 75% of dermatophytic lesions within 1 month.

*Staphylococcus aureus* (Gram-positive), *Escherichia coli* (Gram-negative), and *Candida albicans* (fungal pathogen) were prepared for determining the antimicrobial capacity of ethyl ether and alcoholic extracts of *Prosopis cineraria* leaves [11]. Nutrient broth (10% peptone, 0.5% labanco, 0.5% NaCl, and pH 7.5) was prepared for the growth of *S. aureus* and *E. coli*, while liquid medium (1% peptone, 4% glucose, and pH 5.8) was the growth medium for *C. albicans*. By comparing with standard antibiotics, ethyl ether and alcoholic extracts can inhibit the growth of *S. aureus* (0.80 and 0.74 I/C^a^, respectively), *E. coli* (0.97 and 0.89 I/C^a^, respectively), and *C. albicans* (0.62 and 0.86 I/m^a^, respectively), where I means inhibition zone, a means ratio of diameters of the inhibition zone to extracts (10 µg) under observation (I) and diameter of inhibition zone due to standard reference antibiotics, C means chloramphenicol (30 µg) against *S. aureus* in 30 mm and *E. coli* in 32 mm, and *m* means Mycostatin (100 units) against *C. albicans* in 32 mm [71].

### 4.8. Hypolipidemic and Antiatherosclerotic Efficacy

Hyperlipidemic rabbits can be used as samples to estimate the hypolipidemic and antiatherosclerotic capacity of *Prosopis cineraria* bark extract [11]. High-fat diet and cholesterol powder were fed to rabbits to induce exogenously hyperlipidemic. *Prosopis cineraria* bark extract was used to treat a group of those rabbits, while the other group was treated with a standard drug. The previous research demonstrated that the values of serum total cholesterol, LDL-C, triglyceride, VLDL-C, and ischemic indices (total cholesterol/LDL-C and LDL-C/HDL-C) were decreased after the treatment of 70% ethanol extract of *Prosopis cineraria* bark. Therefore, *Prosopis cineraria* bark has the potential to be used in preventing hyperlipidemic and atherosclerotic [72].

### 4.9. Anti-Inflammatory Activity

Carrageenan- and histamine-induced paw edema in rats was used to demonstrate the anti-inflammatory capacity of *P. juliflora* [3]. Carrageenan, which is comprised of polysaccharides and histamine, was applied in inducing inflammation in rats. The anti-inflammatory capacity of *P. juliflora* was demonstrated by assays of carrageenan-induced and second histamine-induced paw edema [3]. Prostaglandins can promote the second phase of inflammation in rats, and thus, the inhibition of prostaglandins is important for anti-inflammatory drugs. The previous study of SivaKumar et al. [73] displayed that methanol extracts of *P. juliflora* bark can block prostaglandins and inhibited carrageen-induced inflammation in rats, and thus, *P. juliflora* has anti-inflammatory capacity.

### 4.10. Adsorption Potentialities

The adsorption of methyl orange dye from water to leaves and stems of *Prosopis cineraria* can be optimized using simulated waters. The maximum extraction can be gained by changing different physicochemical parameters, including pH, time of equilibration, and sorbent concentrations [74]. The optimized conditions of leaf powder of *Prosopis cineraria* include 89.0% extraction, pH value of 3, 60-minute extraction time, and 0.75 g/500 mL sorbent concentration, which gains maximum extractability of SO_4_^2-^ (20.0%), Cl^−^ (62.0%), NO_3_^−^ (63.0%), Ca^2+^ (64.0%), Fe^2+^ (59.0%), Mg^2+^ (59.0%), etc. [74]. Besides, the optimized conditions of stem powder of *Prosopis cineraria* include 90.5% extraction, pH value of 8, 60-minute extraction time, and 0.5 g/500 mL sorbent concentration, which gains maximum extractability of SO_4_^2-^ (21.0%), Cl^−^ (69.0%), NO_3_^−^ (62.0%), Ca^2+^ (67.2%), Fe^2+^ (61.0%), Mg^2+^ (60.0%), etc. [74].

### 4.11. Antihelminthic Activity

The different solvent extracts of *Prosopis cineraria* stem bark can act as efficient antihelminthic agents in a dose-dependent manner. For example, the methanolic extract of *Prosopis cineraria* stem bark at a dose of 160 mg/mL resulted in paralysis in 25 min and death in 62 min against *Phretima posthuma*, which use a similar time with the standard drug piperazine citrate (10 mg/mL) [75]. Therefore, the extract of *Prosopis cineraria* stem bark has the potential to replace piperazine citrate in antihelminthic properties.

### 4.12. Apoptotic Activity

Breast cancer cell line MCF-7 and noncancerous cell line HBL 100 were used to estimate the apoptotic capacity of methanolic extract of *Prosopis cineraria* leaves [11]. MCF-7 and HBL 100 were stained by different staining methods, including acridine orange/ethidium bromide staining, propidium iodide (Pi) staining, ethidium bromide (Etbr) staining, Hoechst 33342 staining, and DAPI (4′-6′-diamidino-2-phenyl indole) staining [76]. The apoptotic ratio in MCF-7 was increased by the addition of methanolic extract of *Prosopis cineraria* leaves, but that in HBL 100 was not influenced. *Prosopis cineraria* can act as a promising chemotherapeutic agent to treat cancer because it can promote apoptosis or programmed cell death to inhibit the proliferation of cancer cells [76].

### 4.13. Antidepressant Activity

The *Prosopis cineraria* leaf extract can act as an antidepressant agent and skeletal muscle relaxant agent, which is a traditional medicine to treat different CNS diseases [11]. Forced swim test (FST), which was applied for estimating antidepressant effect, showed that the duration of immobility time in FST was reduced by the *Prosopis cineraria* leaf extract at doses of 200 mg/kg [77].

### 4.14. Anticancer Effects

The total alkaloid extractions from leaves of *P. juliflora* have a cytotoxic capacity on cancer cells, such as human T-cell leukemia cells [3]. The IC_50_ values of *P. juliflora* extract at 24, 48, and 72 h were recorded as 90.5, 42.5, and 20.0 *μ*g/ml/1 × 10^6^ cells against cancer cells [78]. Additionally, mitotic cell divisions by chromosome aberrations can be inhibited by extracts of flowers of *P. juliflora* [3]. The root cells of the onion plant *Allium cepa* can be used to describe the antiproliferative capacity of compounds. The compounds obtained from flowers of *P. juliflora* can act as spindle inhibitors and cause the clastogenic effects in *Allium cepa* cells [3].

The anticancer properties of different extracts from *Prosopis farcta* aerial parts to prevent four human tumor cell lines (HepG-2, HeLa, PC3, and MCF-7) were evaluated. The n-butanol extract of *Prosopis* displayed strongest capacity to prevent the MCF-7 cell line (IC_50_ = 5.6 *μ*g/mL), which was comparable to 5-fluorouracil (IC_50_ = 5.4 *μ*g/mL). Besides, the ethyl acetate extract of *Prosopis farcta* also showed the strongest properties against HeLa cell line (IC_50_ = 6.9 *μ*g/mL), while the capacity against 5-fluorouracil was lower (IC_50_ = 4.8 *μ*g/mL) [57].

In contrast, the hydroethanolic extracts of P. *avium* stems and flowers and the infusions of its flowers, which are efficient in antiproliferative, show a significant reduction in cell viability (IC_50_ = 328.74 ± 2.37, IC_50_ = 349.76 ± 0.60, and IC_50_ = 364.79 ± 1.83 µg/mL, respectively) [79].

## 5. Factors Affecting the Pharmacological Activity and Bioactivity

### 5.1. Bioavailability of Phytochemicals in *Prosopis*

Phenolic compounds are the main contributors to the biological activity of *Prosopis*. The oxidation of biomolecules can be inhibited by antioxidants through the donation of hydrogen to free radicals, while this ability of different antioxidants can be determined by 2,2′-diphenyl-2-picrylhydrazyl (DPPH) and 2,2′-azinobis-(3-ethylbenzothiazoline-6-sulfonic acid) (ABTS) assays [4]. In a previous study, the *Prosopis alba* extracts rich in free flavonoids reached SC_50_ values (the scavenging activity of 50% ABTS•+ and DPPH•, and the values are expressed in *μ*g GAE/mL in free phenolic compounds or bound phenolic compounds) between 6 and 17 *μ*g/mL for ABTS, while the results of 50% DPPH scavenging activity were 11 to 15 *μ*g/mL [4]. By comparison, the results gained from *Prosopis alba* dominated by bound phenolic compounds were 2.5 and 7 *μ*g/mL for ABTS and 1.5 and 1.7 *μ*g/mL for DPPH [4]. As a result, free phenolic compounds were less active than bound phenolic compounds. The bound phenolic compounds in *Prosopis* are beneficial for human health because they can avoid the digestion in upper gastrointestinal and be absorbed into blood plasma [81]. Additionally, the free and bound phenolic content in *Prosopis* can protect against the peroxidation of unsaturated biomolecules such as *ß*-carotene and linoleic acid [4]. The free phenolic compounds of *Prosopis* were more active than bound phenolic compounds, which represented that free phenolic compounds are better in protection against lipoperoxidation [4].

The free radical-scavenging activity of the leaves and pods of *Prosopis* was also evaluated in previous research, which could represent the biological activity of *Prosopis*. In *Prosopis tamarugo*, phenethylamine was one of the main aromatic amines, while alkaloids *ß*-phenethylamine and tryptamine were detected in *Prosopis chilensis*. In addition, the total phenolic content was comprised mainly of catechin, which resulted in the observed activity [43].

The extracts of aerial parts of *P. alpataco*, *P. Argentina*, *P. chilensis* (Molina) Stuntz., *Prosopis flexuosa* DC., and *Prosopis pugionata* were applied for isolating the alkaloids tryptamine, phenethylamine, and piperidine in the previous study. The isolated compounds were assessed for DNA binding, *ß*-glucosidase inhibition, and free radical-scavenging (DPPH assay) effects. At a concentration of 0.5 mg/mL, DNA-binding activities showed tryptamine (28%), phenethylamine (0%–27%), and piperidine derivatives (47%–54%), respectively. Tryptamine and 2-*β*-methyl-3-*β*-hydroxy-6-*β*-piperidine dodecanol displayed a moderate inhibition (27%–32%) of the enzyme *ß*-glucosidase at 100 *μ*g/mL. In the DPPH assay, catechin in *Prosopis* showed a free radical-scavenging capacity. HPLC was used to determine the phenolic compounds of the methanol extract of *Prosopis*, which showed the result that more than 70% of phenolic content in *Prosopis* was catechin [82]. The juliflorine from *Prosopis juliflora* existed acetylcholinesterase inhibitory substances [43]. Furthermore, the DPPH and HPLC assays also demonstrated that alkaloids were not the main factor to cause the free radical-scavenging effect of *Prosopis* extracts [82].

Similar to ABTS or DPPH methods, the OH radical inhibition is another method that is based on the single-electron transference (SET) mechanism to evaluate the rate of the OH radical inhibition, and therefore, the antioxidant activity of *Prosopis* gums can be estimated [83]. Due to the higher flavonoid content, the percentage of OH radical inhibition in *Prosopis alba* gum is lower than that of Arabic gum. The principle is that flavonoids can inhibit OH radicals [49]. Metal-chelating activity is also an important secondary antioxidant mechanism that prevents the prooxidant effect of transition metals on the decomposition of hydroperoxides or H_2_O_2_ in the radical chain reaction [84]. A previous study showed that *Prosopis alba* gum has a stronger ability in scavenging ferrous ions than Arabic gum chiefly due to the higher content of tannins, which can chelate with different metal ions [85]. Overall, *Prosopis alba* gum has a stronger antioxidant capacity than Arabic gum, and hence, the applications of *Prosopis* gum as a natural antioxidant are worthy to be further researched [49].

As another antioxidant characteristic, reducing power can describe how the radical reactive species are reduced by a substance in the peroxidation mechanism [86]. Except for polyphenols, reducing power is also influenced by amino acids, reducing sugars, etc. The previous study showed that the reducing power value of Arabic gum was only 1/2 of *Prosopis alba* gum. As the reducing sugar content of Arabic gum was higher than that of *Prosopis alba* gum, the reducing power of *Prosopis alba* gum is mainly attributed to the phenolic compounds [49].

### 5.2. Antinutritional Compounds

Although *Prosopis* seeds have rich nutritional contents, they are not widely used as foods or feeds due to the existence of antinutritional contents [87]. The antinutritional compounds in *Prosopis* include total free phenolics, tannins, phytic acid, L-DOPA, trypsin inhibitor activity, and lectins, while the main antinutritional contents are total free phenolics and tannins, which can reduce protein and fiber digestion and dry matter intake [88]. The total free phenolics and tannins can be excluded by dehulling, soaking, and heat treatment or cooking process due to their water-soluble properties [33].

The antinutritional factors make a negative influence on the nutritional qualities of *Prosopis* [89]. In *Prosopis*, total free phenolic compounds took up about 4.93 to 8.58%, while tannins occurred within 6.81 to 9.15%. The seed coat of *Prosopis* was relatively rich in tannins and total free phenolic compounds. Besides, the protein digestibility of human beings is negatively influenced by the presence of tannins [90].

Several processing methods can avoid the negative influence of antinutritional factors on the edibility of *Prosopis* [91]. As a high-temperature, short, and versatile food operation, extrusion cooking technology can enhance the shelf life and nutritional properties by fully cooking the agricultural raw materials [92]. The extrusion cooking of *Prosopis* can avoid antinutritional factors and develop protein digestibility cost-effectively [15]. The tannin content and lectin agglutinating activity would be increased as the total phenol content and protease inhibitor activity decreased [93]. Furthermore, ruminants may be poisoned by phenolic compounds, which can not only result in lesions in their digestive tract and inhibit the production of digestive enzymes, but also inhibit their digestion of soluble carbohydrates and hemicellulose [94]. During the heat treatment, the formation of complexes between tannins and proanthocyanidins will result in condensation, which can reduce their concentration [93]. Condensed tannins can form tannin-protein complexes, which can prevent microbial degradation of plant proteins, inhibiting their entrance into the small intestine [93].

Autoclaving and dry heat treatment is an advanced technology that can significantly inhibit trypsin inhibitor activity [95]. The main storage form of phosphorus in *Prosopis* is phytic acid, which is a kind of antinutritive factor [96]. The previous research demonstrated that the content of phytic acid of *Prosopis* can be reduced significantly by pressure cooking of soaked-dehulled seeds [96]. Lectins, which are combined with the cells that line the intestinal mucosa, can result in a nonspecific interference with the absorption of available nutrients and reduce the feed intake [90]. Phyto-hemagglutinating activity of *Prosopis* samples was higher with “A” blood group of human erythrocytes but lower with “O” blood group of human erythrocytes, which was consistent with the previous findings of *Mucuna* spp. [95].

### 5.3. Effects of Protein-Phenolic Compound Interactions

Polyphenols and proteins can interact with each other to form nanocomplexes [97]. Protein-polyphenol interactions are noncovalent bonding through hydrophobic, van der Waals, hydrogen bonding, and ionic interactions [98]. Depending on the different strength of the interactions, the complexes formed by polyphenols and proteins can be soluble or precipitate out of solution [99]. Temperature, the structure of the phenolic contents, the properties of the protein, and the existence of other chemical compounds can affect the formation of the protein-polyphenol complex [98]. Besides, the addition of polysaccharides, including Arabic gum, cyclodextrins, pectin, and xanthan gum, can increase solubility and avoid sedimentation of protein-polyphenol complex [98]. A previous study used fluorescence measurements to demonstrate that a pea (*Pisum sativum*) protein isolate would interact with quercetin via hydrophobic interactions and hydrogen bonding and the PPI-Q-MG (pea protein isolate-quercetin-mesquite gum) can develop the physical and chemical stability of quercetin [98]. As a result, the interactions of protein-phenolic compounds contribute to developing the transport systems of quercetin from *Prosopis* food products and beverages.

## 6. Conclusion and Future Direction

In conclusion, *Prosopis* are underutilized legume plants that are widely distributed in arid, semiarid, tropical, and subtropical countries. *Prosopis* can be used as food, beverages, firewood, timber, livestock feed, vegetable, construction and fencing material, medicine, and shade. The chemical constituent of *Prosopis* includes macronutrients (carbohydrate, protein, and fat), polyphenolic compounds, amino acids, fatty acids, minerals, fiber, carotenoids, and ascorbic acid. *Prosopis* flour has high protein and low carbohydrate and fat content, which can contribute to a healthy diet. *Prosopis* contain essential amino acids, such as isoleucine and valine, which do not exist in other grains. *Prosopis* can also be a good source of linoleic acid and minerals including potassium, magnesium, calcium, and phosphorus. Furthermore, *Prosopis* contains abundant phenolic compounds, such as flavonoids (especially *C*-glycosyl flavonoids), tannins, 4′-O-methyl-gallocatechin, mesquitol, and quercetin O-glycosides, but the content of hydrolysable tannins and anthocyanins is relatively low. Phenolic compounds are the most valuable content in *Prosopis* as they significantly contribute to the pharmacological value and nutraceutical potential in *Prosopis*. *Prosopis* has different pharmacological functions that can be divided into antioxidant activity, antihyperglycemic activity, analgesic and antipyretic activity, antitumor activity, respiratory and gastrointestinal activity, anticonvulsant activity, antibacterial activity, hypolipidemic and antiatherosclerotic efficacy, anti-inflammatory activity, adsorption potentialities, antihelminthic activity, apoptotic activity, antidepressant activity, and anticancer effects. Except for the high content of phenolic compounds, *Prosopis* can also be a good source of protein, galactomannans, and dietary fiber. The moisture content in *Prosopis* is relatively low, which helps to increase the shelf life of *Prosopis* products. However, the factors influencing the pharmacological activity and bioactivity in *Prosopis* are of concern, such as bioavailability of phytochemicals, antinutritional compounds, and effects of protein-phenolic compound interactions.

In the future, as a rich source of phenolic compounds, the structures and properties of phytochemicals in *Prosopis* necessitate further studies. In addition, it is vital to develop processing methods to maintain the nutritional compounds of *Prosopis*, when it is used as food. Furthermore, when *Prosopis* is grown, the use of pesticides should be strictly controlled, while the chemical compounds and nutritional properties affected by genetic diversity and different living conditions are worthy to be further researched. Finally, *Prosopis* has great potentiality to be applied in medicine, but it is still necessary to study the appropriate standards and reasonable dosage of different varieties of *Prosopis*.

## Figures and Tables

**Table 1:** Nutritional composition of *Prosopis* [16, 38].

Nutrient	Unit	*P. pallida* (value per 100 g)	*P. africana* (value per 100 g)
Proximate composition			
Crude protein	G	9.1	23.6
Fiber	G	18.4	3.3
Ash	G	3.9	4.4
Carbohydrate by difference	G	69.2	54.0
Energy	kJ	1530	1792.8
Sodium (Na)	Mg	110	110.7
Potassium (K)	Mg	2650	617.5
Calcium (Ca)	Mg	80	362.5
Magnesium (Mg)	mg	90	1420.1
Copper (Cu)	mg	Trace	46.2
Zinc (Zn)	mg	Trace	22.4
Manganese (Mn)	mg	Trace	36.2
Iron (Fe)	mg	30	15.5
Aspartic acid (Asp)	g	8.51	10.0
Serine (Ser)	g	4.96	3.2
Glutamic acid (Glu)	g	10.07	13.3
Proline (Pro)	g	23.40	3.0
Glycine (Gly)	g	4.68	3.3
Alanine (Ala)	g	4.26	2.8
Methionine (Met)	g	0.57	1.4
Valine (Val)	g	7.80	4.2
Isoleucine (Ile)	g	3.26	3.7
Leucine (Leu)	g	7.94	6.4
Tyrosine (Tyr)	g	2.84	3.2
Lysine (Lys)	g	4.26	4.2
Histidine (His)	g	1.99	2.5
Arginine (Arg)	g	4.82	5.0

**Table 2:** Methods for the *determination* of chemical components of *Prosopis spp.*

Chemical components	*Prosopis* Species	Plant part	Methods	Major findings	Reference
Polyphenol	*P. laevigata*	Leaves	Aqueous acetone extraction; purified fractions; HPLC	Gallocatechin, coumaric acid, morin, rutin, catechin, gallic acid, naringenin, epicatechin gallate, and luteolin are the main phenolic compounds.	[2]
Carbohydrate	*P. nigra*	Pods	Phenol-sulfuric acid method; Somogyi–Nelson method	The carbohydrate content of aqueous extraction is higher than alcoholic extraction. The main carbohydrate is sucrose, and the content of glucose and fructose is low.	[1]
Protein	*P. nigra*	Pods	Bovine serum albumin (BSA) standard	Total protein content was 4.2%.	[1]
Polyphenol	*P. nigra*	Pods	Folin–Ciocalteu; aluminium chloride colorimetric method; 2% ferric ammonium sulfate in 2 N HCl	0.18–0.41 g GAE/100 g DW (free phenolic content); 0.07–0.13 g QE/100 g DW (flavonoid content); 4.64 to 6.90 g QTE/100 g DW (proanthocyanidin content).	[1]
Phytic acid	*P. nigra*	Pods	Addition of Wade reagent	Phytic acid concentrations were 1.19%.	[1]
Polyphenol	*P. alba*	Exudate gum	UPLC-ESI-TOF/MS	Ferulic, coumaric, and caffeic acids are significant phenolic contents in *Prosopis* gum.	[49]
Polyphenol	*P. alba*	Seeds	RP-HPLC-DAD; MALDI-TOF MS analysis; Nanoflow HPLC-ESI-MS/MS analysis	6-*C*-pentosyl-8-*C*-glucosyl apigenin (isoschaftoside), 6-*C*-glucosyl-8-*C*-pentosyl apigenin (schaftoside), apigenin 6-*C*-(6″-*O*-glycosyl) glycosyl-8-*C*-glycoside, and 8-*C*-(6″-*O*-glycosyl) glycosyl-6-*C*-glycoside were determined.	[26]
Polyphenol	*P. nigra*	Pods	HPLC-DAD; HPLC-ESI-MS/MS; NMR analysis	The bound flavonoids take up 89% of total flavonoids; the higher content of anthocyanins results in the darker color of *Prosopis.*	[4]
Macronutrients	*P. alba*	Cotyledons	The Association of Official Analytical Chemists (AOAC, 2000) methods	8.97 ± 0.05 g/100 g (carbohydrate content); 0.21 ± 0.07 g/100 g (soluble reducing sugar content); 12.20 ± 0.05 g/100 g flour (fat content); albumin and globulin were the major proteins.	[31]
Amino acids	*P. alba*	Cotyledons	Biochrom 30 Series Amino Acid Analyzer	*Prosopis alba* contains amino acids with sulfur, which are limited in other grains.	[31]
Minerals	*P. alba*	Cotyledons	Quadrupole inductively coupled plasma mass spectrometry	Potassium (K) content is high (7.5 mg/g), and sodium (Na) content is low (0.09 mg/g).	[31]
Fatty acids	*P. alba*	Cotyledons	Agilent Technologies (Model 6890N) GC with flame ionization detector	Unsaturated fatty acids (PUFAs) are the major fatty acids.	[31]
Phenolic compounds	*P. chilensis*	Mesocarp	HPLC-DAD-MS/MS	Common constituents were flavone C-glycosides.	[53]
Carbohydrate	*P. flexuosa*	Seed	Gas chromatography characterization (GC)	Galactose and mannose are the main carbohydrates.	[32]
Flavonoids	*P. laevigata*	Seed	Reversed-phase high-performance liquid chromatography (RP-HPLC)	The main flavonoid was apigenin.	[15]

**Table 3:** Identification of phytochemicals in different *Prosopis spp.*

Species and plant parts	Phytochemicals	References
*Prosopis laevigata* leaves	Gallic acid, coumaric acid, catechin, gallocatechin, epicatechin gallate, rutin, morin, naringenin, luteolin	[2]

*Prosopis alba* exudate gum	Ferulic acid 4-glucuronide, ferulic acid rhamnosyl-hexoside, ferulic acid, coumaric acid, esculetin derivative, 7-*O*-methylapigenin, apigetrin, chrysin, 3-galloylquinic acid, caftaric acid, chlorogenic acid, chlorogenic acid, *p*-coumaroylquinic acid, valoneic acid dilactone, digallic acid, kaempferol 3-*O*-arabinoside	[49]

*P. alba* seed germ flour	Apigenin 6-C-(6″-O-glc) arab-8-C-glc, apigenin 6-C-glc-8-C-(6″-O-glc) Arab, apigenin 6-*C*-glc-8-*C*-glc, apigenin 5,7-*O*-diglucosides, apigenin 6-*C*-pentoside-7-*C*-hexoside, apigenin 6-*C*-arab-8-*C*-glc, apigenin 6-*C*-glc-8-*C*-arab	[26]

*Prosopis cineraria* pods	3-Benzyl-2-hydroxy-urs-12-en-28-oic acid, maslinic acid-3 glucoside, linoleic acid, prosophylline, 5,5′-oxybis-1,3-benzenediol, 3,4,5-trihydroxycinnamic acid 2-hydroxyethyl ester, 5,3′,4′-trihydroxyflavanone 7-glycoside	[12]

*Prosopis nigra* pods	Cyanidin rhamnosyl hexoside, cyanidin-3-hexoside, peonidin-3-hexoside, malvidin dihexoside, cyanidin malonyl hexoside, petunidin-3-hexoside, malvidin rhamnosyl hexoside, malvidin-3-hexoside, apigenin-di-*C*-hexoside, quercetin-dihexoside rhamnoside, vitexin, isovitexin	[4]

*Prosopis alba* cotyledons	Isoschaftoside hexoside, schaftoside hexoside, vicenin II/isomer, isoschaftoside, schaftoside, vitexin, isovitexin	[31]

*Prosopis chilensis* mesocarp flour	Cyanidin 3-hexoside, peonidin 3-hexoside, cyanidin malonyl hexoside, peonidin malonyl hexoside, ellagic acid hexoside, hydroxyferulic acid hexoside, vicenin II/isomer, schaftoside, quercetin-dihexoside, quercetin-hexosidepentoside, quercetin-methyl ether rhamnoside hexoside, quercetin-rhamnoside-hexoside, isovitexin, quercetin-methyl ether rhamnoside hexoside	[53]

*Prosopis farcta* aerial parts	Tetradecane, pentadecane, dodecanoic acid, 1-tridecene, 4-methyl-14-pentadecenoic acid, 1-icosene, octadecane, nonadecane, pentadecanoic acid, nonadecanoic acid	[54]

**Table 4:** Biological activities of compounds of different *Prosopis spp*.

Bioactivity	Species	Experiment/Model	Dosage/Formulation	Result	Reference
Antioxidant	*Prosopis laevigata* leaves	DPPH	100 *μ*L sample was mixed with 2900 *μ*L of DPPH solution.	The EC_50_ for DPPH radical-scavenging capacity by acetone crude extracts was about 3000 ppm.	[2]
	*Prosopis laevigata* leaves	Hydroxyl radical scavenging	100 *μ*L each of deoxy-d-ribose (28 mM), FeCl_3_ (1 mM), EDTA (1.04 mM), H_2_O_2_ (1 mM), and ascorbic acid (1 mM) with 500 *μ*L of diluted sample.	The IC_50_ for hydroxyl radical-scavenging capacity by acetone crude extracts was about 1588 ppm.	[2]

Cardioprotection	*Prosopis laevigata* leaves	Inhibition of LDL oxidation	The reactant ingredients consisted of 700 *μ*L of PBS, 100 *μ*L of CuSO4 (0.5 mM), 100 *μ*L of LDL, 100 *μ*L of samples or standards.	Purified extracts from *Prosopis* can avoid the generation of peroxide radicals from the oxidized LDLs.	[2]

Antihyperglycemic activity	*Prosopis laevigata* leaves	Angiotensin-converting enzyme (ACE) inhibition	The spectrometric procedure by [80].	The oligomeric polyphenols in *Prosopis* can inhibit the angiotensin enzymes I and II.	[2]
	*Prosopis cineraria* stem bark	Alloxan-induced hyperglycemia model	Hyperglycemic mice were fed a dose of 300 mg/Kg B W once a day for 1.5 months.	Bodyweight and fasting blood glucose level of mice were decreased, while liver glycogen content developed.	[11]

Analgesic and antipyretic activity	*Prosopis cineraria* stem bark	Brewer's yeast-induced hyperpyrexia model in experimental rats	The ethanolic extract was estimated by tail immersion and hot plate method. The aqueous extract was estimated by acetic acid-induced writhing test mode.	The extract of *Prosopis* displayed analgesic and antipyretic capacity.	[61]

Antitumor activity	*Prosopis cineraria* leaves and bark	Male Wistar rats	Protective action against induced experimental liver tumors.	The administration of the extract (200 and 400 mg/kg) reduces mitochondrial lipid peroxidation (LPO) and liver weight.	[7]

Anti-inflammatory capacity	*Prosopis nigra* pods flour	Cyclooxygenase inhibition studies	*Prosopis* extract inhibits the conversion of arachidonic acid (AA) to prostaglandin H_2_ (PGH_2_) by human recombinant cyclooxygenase 2 (COX-2).	0.66 ± 0.03 *μ*g GAE/mL of *P. nigra* crude extracts can inhibit 50% enzyme activity (IC_50_).	[4]
	*P. juliflora* bark	Carrageenan- and histamine-induced paw edema in rats.	Inflammation by carrageenan, histamine, and prostaglandins.	Methanol extracts of *P. juliflora* bark show anti-inflammatory capacity.	[3]

Antibacterial activity	*Prosopis cineraria* stem bark	Agar well diffusion method	Four Gram-negative bacteria, (*E. coli*, *P. aeruginosa*, *S. typhi*, and *K. pneumoniae*) are used to estimate the antibacterial activity of *Prosopis*.	*Prosopis cineraria* showed antibacterial activity at 250 *μ*g/mL.	[11]
	*Prosopis cineraria* leaves	Agar well diffusion method	Three microorganisms *Staphylococcus aureus* (Gram-positive), *Escherichia coli* (Gram-negative), and *Candida albican*s (fungal pathogen) are used.	*Prosopis cineraria* display positive reactions against all these microorganisms.	[71]

Anticonvulsant activity	*Prosopis cineraria* stem bark	Maximal electroshock (MES) and pentylenetetrazole (PTZ)-induced convulsions in mice	Protect against hind limb tonic extensions (HLTE) induced by MES and PTZ-induced seizures.	*Prosopis cineraria* demonstrated anticonvulsant activity at doses of 200 and 400 mg/kg and phenytoin (25 mg/kg).	[11]

Hypolipidemic and antiatherosclerotic efficacy	*Prosopis cineraria* stem bark	Hyperlipidemic rabbits	Rabbits were fed by high-fat diet and cholesterol powder.	70% extract of *Prosopis cineraria* stem bark showed hypolipidemic activity.	[11]

Antihelminthic activity	*Prosopis cineraria* stem bark	Indian earthworm	Estimation of time of paralysis (*p*) and time of death (d) of the worm.	The extract at a dose of 160 mg/mL caused paralysis in 25 min and death in 62 min.	[75]

Apoptotic activity	*Prosopis cineraria* leaves	Breast cancer cell line MCF-7 and noncancerous cell line HBL 100	Giemsa, ethidium bromide, propidium iodide, and Hoechst are used to stain.	*Prosopis cineraria* leaves can inhibit the proliferation of MCF-7 breast cancer cells.	[76]

Antidepressant activity	*Prosopis cineraria* leaves	Forced swim test (FST)	Compared with imipramine (15 mg/kg. p.o.).	*Prosopis cineraria* leaves show antidepressant activity at doses of 200 mg/kg.	[11]

Anticancer activity	*Prosopis farcta* aerial parts	Against four human tumor cell lines	HepG-2, HeLa, PC3, and MCF-7.	Extract against MCF-7 cell line (IC_50_ = 5.6 *μ*g/mL) and HeLa cell line (IC_50_ = 6.9 *μ*g/mL).	[57]

## Data Availability

The data supporting this review are available from previously reported studies and datasets, which have been cited. The processed data are available from the corresponding author upon request.
